# Age Differences in Aortic Stenosis

**DOI:** 10.31083/RCM28185

**Published:** 2025-04-17

**Authors:** Tomoyo Hamana, Teruo Sekimoto, Aloke V. Finn, Renu Virmani

**Affiliations:** ^1^CVPath Institute, Inc, Gaithersburg, MD 20878, USA; ^2^School of Medicine, University of Maryland, Baltimore, MD 21201, USA

**Keywords:** aortic stenosis, calcific aortic valve disease, bioprosthetic valve failure

## Abstract

Aortic stenosis (AS) is a significant and growing concern, with a prevalence of 2–3% in individuals aged over 65 years. Moreover, with an aging global population, the prevalence is anticipated to double by 2050. Indeed, AS can arise from various etiologies, including calcific trileaflets, congenital valve abnormalities (e.g., bicuspid and unicuspid valves), and post-rheumatic, whereby each has a distinct influence that shapes the onset and progression of the disease. The normal aortic valve has a trilaminar structure comprising the fibrosa, spongiosa, and ventricularis, which work together to maintain its function. In calcific AS, the disease begins with early calcification starting in high mechanical stress areas of the valve and progresses slowly over decades, eventually leading to extensive calcification resulting in impaired valve function. This process involves mechanisms similar to atherosclerosis, including lipid deposition, chronic inflammation, and mineralization. The progression of calcific AS is strongly associated with aging, with additional risk factors including male gender, smoking, dyslipidemia, and metabolic syndrome exacerbating the condition. Conversely, congenital forms of AS, such as bicuspid and unicuspid aortic valves, result in an earlier disease onset, typically 10–20 years earlier than that observed in patients with a normal tricuspid aortic valve. Rheumatic AS, although less common in developed countries due to effective antibiotic treatments, also exhibits age-related characteristics, with an earlier onset in individuals who experienced rheumatic fever in their youth. The only curative therapies currently available are surgical and transcatheter aortic valve replacement (TAVR). However, these options are sometimes too invasive for older patients; thus, management of AS, particularly in older patients, requires a comprehensive approach that considers age, disease severity, comorbidities, frailty, and each patient’s individual needs. Although the valves used in TAVR demonstrate promising midterm durability, long-term data are still required, especially when used in younger individuals, usually with low surgical risk. Moreover, understanding the causes and mechanisms of structural valve deterioration is crucial for appropriate treatment selections, including valve selection and pharmacological therapy, since this knowledge is essential for optimizing the lifelong management of AS.

## 1. Introduction

The prevalence of clinical aortic stenosis (AS) increases with age, affecting approximately 2–3% of individuals >65 years of age and rising to 7% in those >80 years [1, 2, 3]. With increasing life expectancy and an aging population, the prevalence of AS is growing worldwide [4]. AS is the most prevalent type of valvular heart disease in developed countries and is becoming an ever-increasing public health burden [5, 6]. Furthermore, the number of elderly patients with calcific AS is expected to more than double over the next 30 years in both Europe and the United States [7]. Nonetheless, there are no effective medical therapies, and the only available therapeutic options are invasive procedures: surgical aortic valve replacement (SAVR) or transcatheter aortic valve replacement (TAVR).

The etiology of AS is diverse, with varying epidemiological and histopathological features, molecular mechanisms, and optimal treatment strategies. Therefore, a deeper understanding based on its different etiologies is essential for addressing this increasingly prevalent disease, which poses a significant global health burden.

In this review, we summarize the current understanding of the mechanisms underlying the natural progression of calcific aortic valve disease. We then provide a detailed description of the epidemiological, histopathological, and molecular mechanisms of calcific, congenital, and rheumatic AS individually. Lastly, we discuss the lifetime management of AS, incorporating our histopathological insights into bioprosthetic valve dysfunction.

## 2. Normal Anatomy and Histological Structure of the Aortic Valve 

**Fig. 1.**
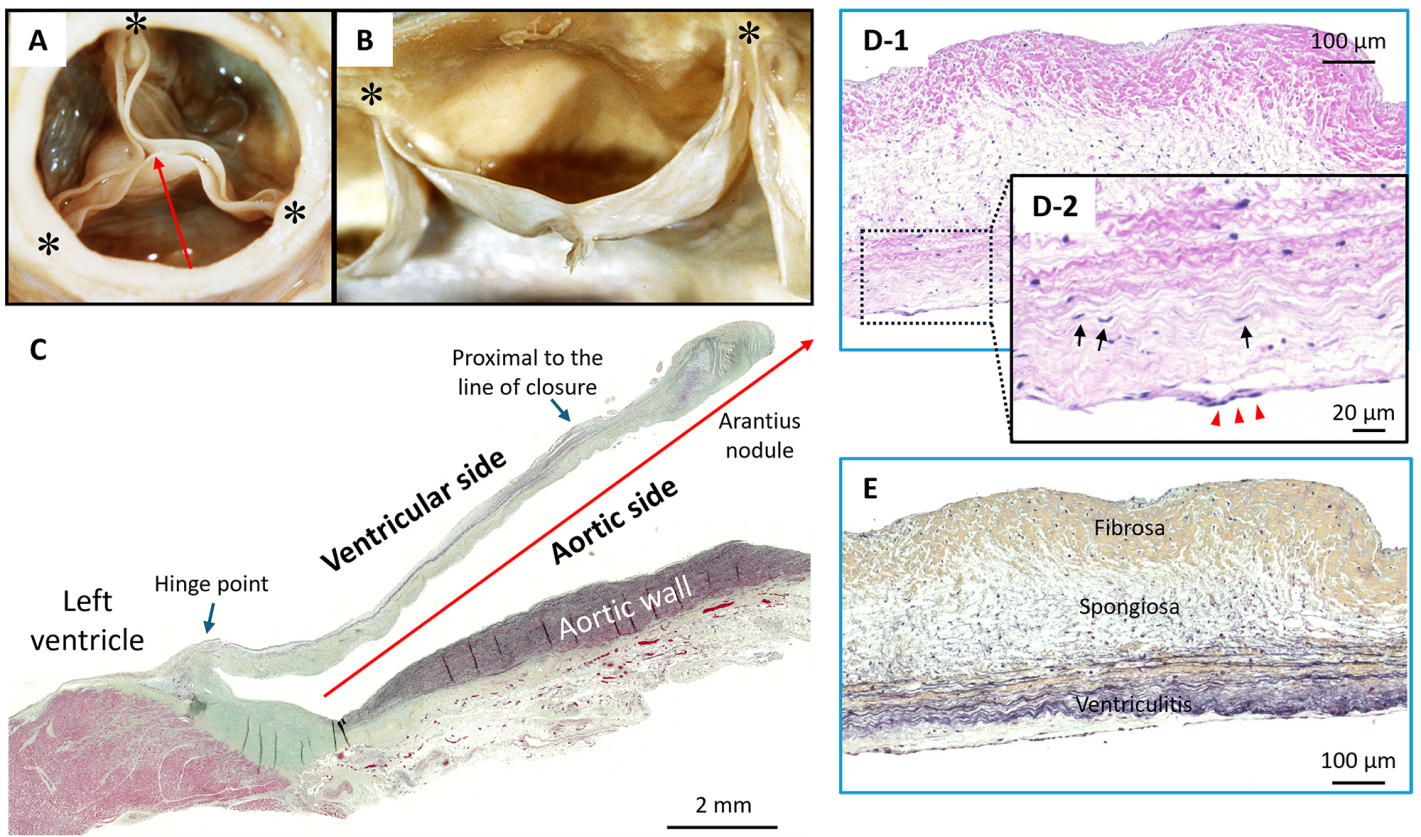
**Representative Histology Images of a Normal Tricuspid Aortic Valve**. (A) Gross image of a normal aortic valve from the aortic surface and (B) lateral surface. Asterisks (*) show commissures. The sinus of Valsalva of the non-coronary leaflet and the free margin of the leaflet are shown, along with the line of closure and the nodule of Arantius. (C) Normal aortic valve of 48-year-old male who experienced sudden death from an unknown cause, scale bar: 2 mm. Low-power image of left coronary cusp leaflet (Movat Pentachrome stain). Red arrow shows the dimension of the slice corresponding to (A). The hinge point and area proximal to the line of closure (blue arrows) are the regions prone to greater mechanical forces. (D,E) Normal aortic valve images of 23-year-old male who died with seizure. Low-power image (D-1, scale bar: 100 µm) and high-power image (D-2, scale bar: 20 µm) of H&E stains show valve interstitial cells comprising aortic valve (black arrows) and valve endothelial cells covering valve layers (red triangles). (E) Low-power image of Movat Pentachrome stain shows three layers of lamina fibrosa (upper), spongiosa (middle), and ventricularis (lower), scale bar: 100 µm. H&E, Hematoxylin and Eosin. Images (A) and (B) are reproduced with permission from Virmani R *et al*. Cardiovascular pathology (pp. 248). 2nd edn. W.B. Saunders Company: Philadelphia. 2001 [9].

A normal aortic valve apparatus is located in the aortic root, which is anatomically defined as the section of the thoracic aorta extending from the sinotubular junction to the basal ring (aortic annulus). The aortic root, approximately 2 to 3 cm in length, comprises three main components: the three aortic valve leaflets, the sinus of Valsalva (the expanded portion of the aortic root), and the three interleaflet triangles (Fig. 1A,B) [8, 9]. The coronary ostia are typically located in the left and right coronary sinuses. The aortic valve has three semicircular cusps and leaflets (left, right, and non-coronary), which are attached to the aortic sinus in a ring-like structure (semi-lunar attachment) and are cornet-shaped, called the annulus fibrosus (surgical aortic annulus). For the interventionist, the annulus, which means “ring”, is the ventriculoaortic junction. The geometric height of the leaflet is different from the effective height of the leaflet [10]. The three leaflets are uneven; the free margin length varies (right 35.2 ± 4.1 mm, range 27.6 to 46.4 mm; left 32.6 ± 3.8 mm, range 25.8 to 43.7; and non-coronary 34.2 ± 4.3, range 26.4 to 47.8 mm). The aortic cusps are thin and translucent with a thickness of under 1 mm. The “hinge” portion of the cusps is located at the ventriculoaortic junction, and is susceptible to greater mechanical stress (Fig. 1C) [11]. This is usually the site where fibrosis is observed, typically by 20 years of age. The line of closure of the leaflet is located away from the free margin of the leaflet and is typically the first site of calcification (Fig. 1C).

Histologically, each leaflet consists of three layers: lamina fibrosa, spongiosa, and ventricularis (Fig. 1D,E). The aortic and ventricular surfaces are covered by valvular endothelial cells (VECs), which work as the interface between the blood and the leaflet. Valvular interstitial cells (VICs) are quiescent, fibroblast-like cells present throughout these three layers and are the major cell component of the valve leaflet. The fibrosa layer, located on the aortic side, consists largely of type I and type III fibrillar collagen with dispersed VICs, which are thought to reinforce the valvular structure [12, 13]. The middle spongiosa layer is composed primarily of proteoglycans and glycosaminoglycans, which absorb a portion of the mechanical stress generated during the cardiac cycle. The ventricularis layer, localized on the ventricular inflow side, consists of collagen and elastin fibers [14, 15].

The aortic valve is structured to allow low-impedance, unidirectional forward blood flow during opening, and to close with enough strength to endure systemic blood pressure [16]. During systole, the aortic valve experiences laminar shear stress on the ventricular side as blood flows past the leaflets, while during diastole, oscillatory shear stress acts on the aortic side as blood pools into the sinuses [17]. Diastolic coronary flow partially generates laminar shear stress on the left and right cusp, while the non-coronary cusp may be solely exposed to oscillatory shear stress. Therefore, throughout the cardiac cycle, the ventricularis layer is subjected to higher, unidirectional forces due to blood flow, while the fibrosa layer experiences lower, bidirectional wall shear stress, especially on the non-coronary leaflet [18, 19].

The earliest change occurs on the aortic surface and is the result of stress-induced cellular senescence, which includes endothelial barrier dysfunction and allows blood lipids to enter the subendothelial space. The mechanical stress pattern generated by the blood flow over time likely initiates aortic valve sclerosis, primarily affecting the aortic side of the valve, typically beginning at the base of the leaflet. This, like the atherosclerotic process, may be followed by inflammatory infiltrate, cell death, and eventual calcification.

## 3. Etiology and Epidemiology of Aortic Stenosis

AS is caused by three main etiologies: calcific AS (previously referred to as degenerative or senile AS), congenital abnormalities such as bicuspid or unicuspid valve, and rheumatic AS [11]. Table 1 (Ref. [11]) shows the frequency of each AS etiology among patients who underwent SAVR.

**Table 1.** **Etiology of Surgically Removed Aortic Valves**.

Etiology		Mayo Clinic	University of Minnesota	London	Mayo	AFIP	Baylor University	Toronto
		(1965)	(1979–1983)	(1976–1979)	(1990)	(1990–1997)	Medical Center	(2008)
							(1993–2004)	
Tricuspid degenerative (calcific aortic stenosis)		0%	28%	12%	51%	49%	46%	64%
Congenital								
	Bicuspid	49%	49%	56%	36%	30%	49%	32%
	Unicuspid	10%	1%	0%	0%	6%	4%	3%
Rheumatic		33%	23%	24%	9%	13%	-	11%
Other		7%	0%	8%	2%	2%	1%	1%

Modified with permission from Ladich E *et al*. Future Cardiol 2011; 7: 629–642 [11]. AFIP, armed forces institutee of pathology.

Unicuspid aortic valve (UAV), has 2 subtypes that are associated with valve dysfunction, one found in childhood and adolescence (under the age of 25 years), and the second in adulthood (mean age 51 ± 14 years). UAV is uncommon, affecting only 0.02% of the population [20], and accounts for only 4–5% of patients who have undergone SAVR [21, 22]. In contrast, bicuspid aortic valve (BAV) occurs in 0.5–2% of the general population [23, 24] and accounts for 20–30% of patients who underwent SAVR [25, 26]. Both UAV and BAV are more common in men than women (UAV: male:female = 4:1, BAV: male:female = 1.4–4:1) [23, 27].

Calcific AS in patients with tricuspid aortic valve (TAV) occurs in approximately 2–3% of those 65 and over, as well as 7% of those over 80 years old [1, 2, 3]. Overall, calcific AS is more prevalent in men than women (male:female = 1.6:1) [11]. The rarest form is quadricuspid valve, with an estimated incidence of 0.013–0.043% in the general population. The most common clinical manifestation is aortic regurgitation (75%), and pure AS is seen only in 0.7% of cases [28, 29]. A histopathological study from Toronto General Hospital involving over 1000 consecutive surgically excised aortic valves found that TAV, BAVs, and UAVs were present in 64.5%, 31.9%, and 3.0% of patients, respectively [30]. Rheumatic heart disease was present in 11% of all cases, which is lower than earlier studies, indicating that the prevalence of post-rheumatic AS has declined over the past half-century in developed countries.

The burden of calcific AS is expected to increase in the coming decades, due to the aging population and the lack of effective prevention strategies. Current prevalence data and demographic projections indicate that the number of patients over 70 years old with calcific AS will double to triple over the next 50 years in developed countries [7, 31].

## 4. Histopathology of Calcific Aortic Stenosis

Calcific AS is a progressive disease, characterized by pathological features that progress from minimal fibrocalcific changes in early lesions to fibrotic thickening and calcium nodules in advanced stages. Our laboratory has observed varying degrees of calcification in valves removed during both surgery and autopsy.

**Fig. 2.**
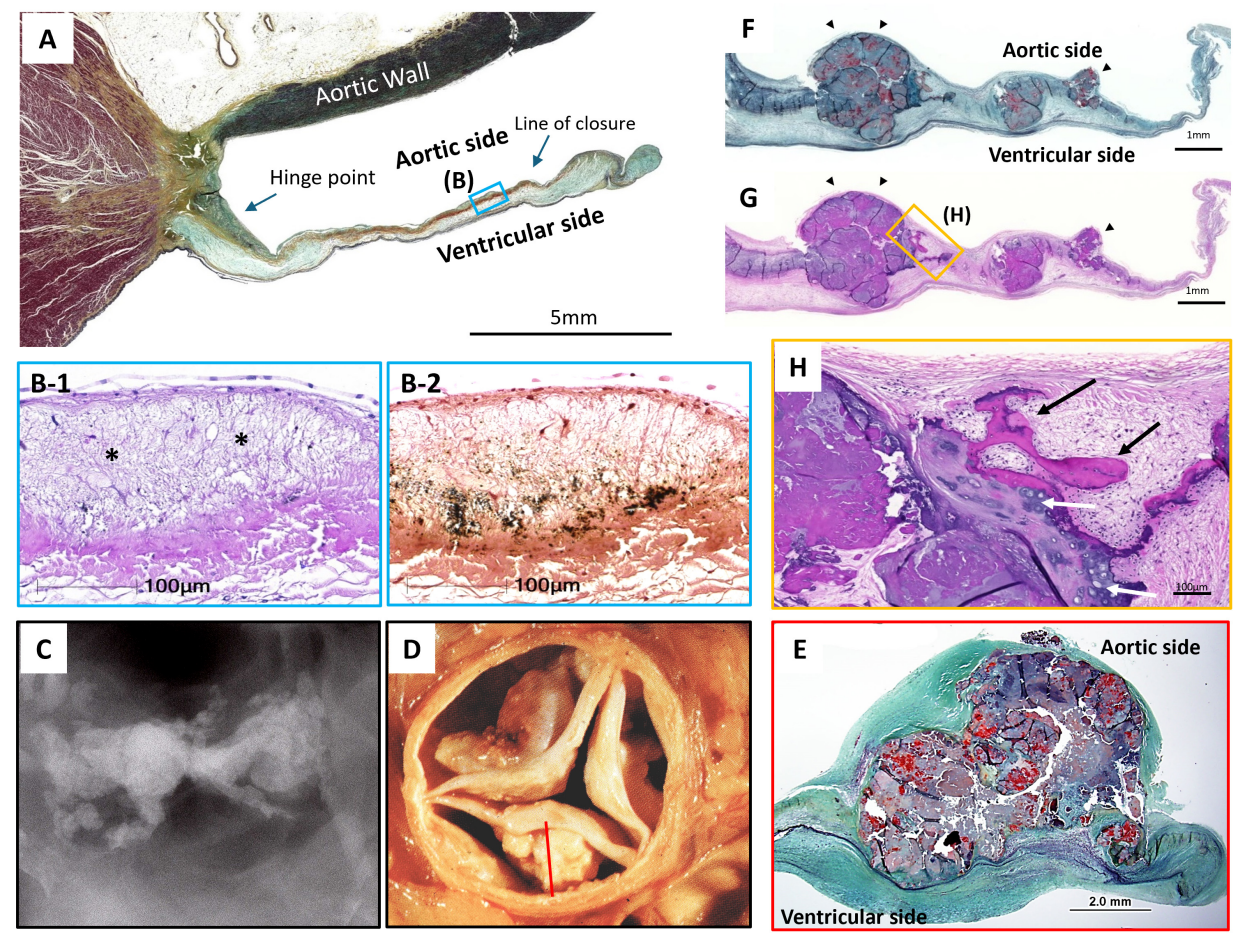
**Representative Histology Images of Tricuspid Aortic Valve Calcification**. (A,B) Early-stage calcification resembling atherosclerotic changes. Aortic valve leaflet from 47-year-old male who died due to accidental head trauma. scale bar: 5 mm. (A) Low-power image of the left coronary cusp leaflet stained with Movat Pentachrome (B1,B2). High-power images from the blue box in (A) at the line of closure, showing lipid insudation (*). (B1) H&E revealing calcification as black dots. (B2) von Kossa stain highlighting calcific deposits, scale bar: 100 µm. (C–E) Advanced calcification in aortic valves. Aortic valve from an 82-year-old female. (C) Radiograph showing severely calcified leaflets. (D) Gross image showing significant nodular calcification within the cusps, with large deposits filling the sinuses. (E) Histologic section of the cusp revealing calcific nodules disrupting the normal architecture of the leaflet, scale bar: 2.0 mm. Note fibrotic thickening of the ventricular surface. (F–H) Bone formation in advanced aortic stenosis. Aortic valve leaflet from a 70-year-old female who underwent surgical aortic valve replacement (SAVR) for severe aortic stenosis (AS), F: scale bar: 1 mm; G: scale bar: 1 mm; H: scale bar: 100 µm. Low-power images of Movat Pentachrome (F) and H&E (G) stain showing nodular calcification on the aortic surface (black arrow). The upper and lower surfaces indicate the aortic and ventricular side, respectively. (H) High-power H&E stain image from the boxed region in (G), revealing ossification (black arrow) and cartilaginous metaplasia (white arrow) at the edge of nodular calcification. H&E, Hematoxylin and Eosin. Modified and reproduced with permission from Sato Y *et al*. Mastering Structural Heart Disease (pp.12). 1st edn. Wiley: NJ. 2023 (A, B1, B2, F, G, and H) [32] and Elena L *et al*., Future Cardiol 2011; 7: 629–642 (C, D, and E) [11].

**Fig. 3.**
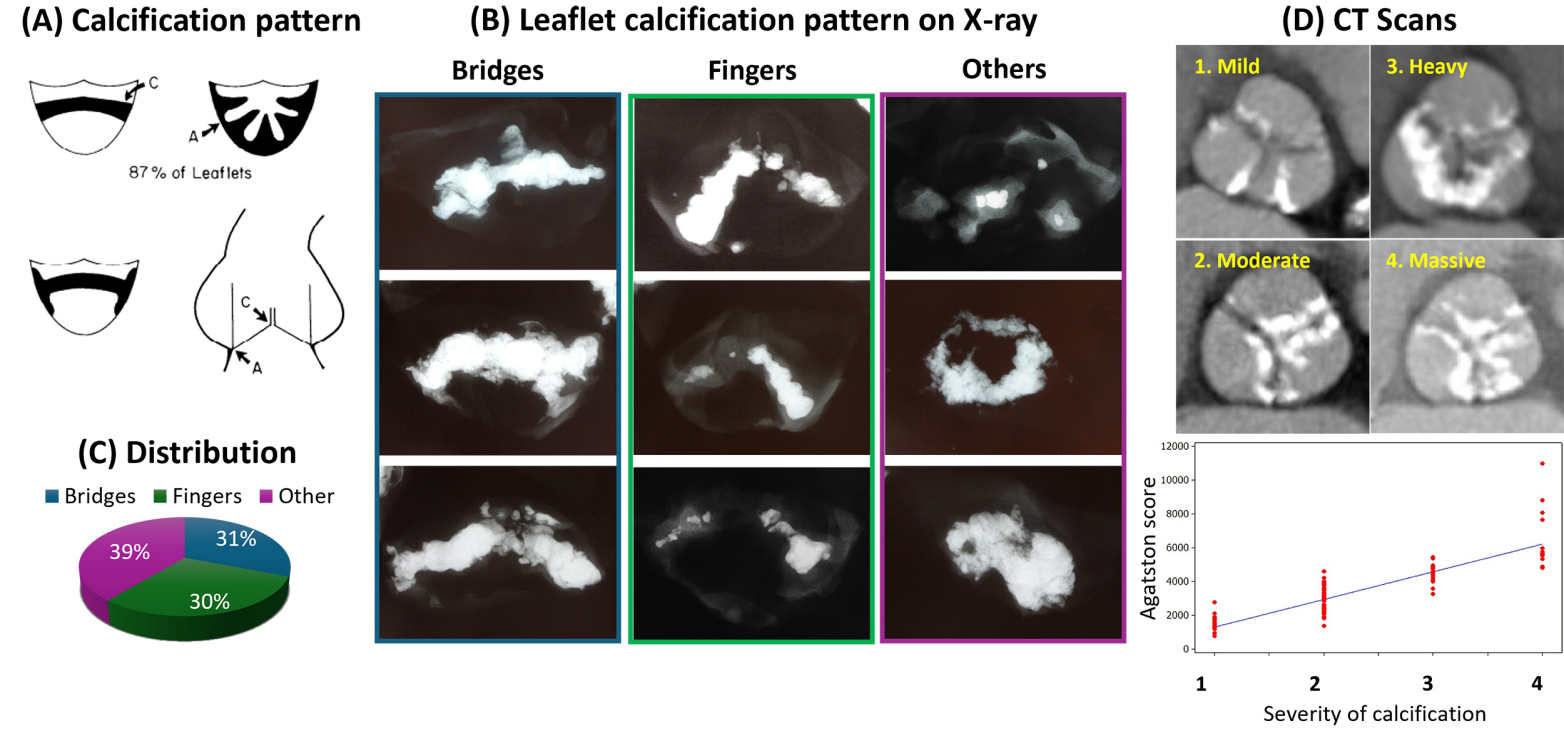
**Calcification Patterns Seen in Radiography and Computed Tomography**. (A) Patterns of calcific deposit and regions of cusp flexion. (Upper left) Coaptation pattern: calcification predominantly occurs along the coaptation line (C) of the leaflet. (Upper right) Radial pattern: calcium accumulates in spokes radiating outward from the cusp attachment area (A) toward the center of the cusp. (Lower left) Combination pattern: calcification occurs along both the coaptation line and the cusp attachment, a frequently observed phenomenon. (Lower right) As the aortic valve opens and closes, the cusps of the valve experience significant flexion at the cusp attachment area (A). The cusps also undergo flexion along the coaptation line (C). (B) Patterns of leaflet calcification seen in radiograph. Bridge forms occur as two spokes along the line of cusp coaptation, corresponding to the coaptation pattern in (A), whereas finger forms are seen when calcium deposits incompletely along the line of cusp coaptation. (C) Among our autopsy calcific aortic stenosis cases, 31% accounted for bridge forms, 30% for finger forms, and 39% for other forms. (D) Different grades of calcification are seen in computed tomography: (1) mild, (2) moderate, (3) heavy, and (4) massive calcification, which shows a significant positive correlation with the Agatston score. CT, computed tomography. Modified and reproduced (A) with permission from Thubrikar MJ *et al*. Am J Cardiol 1986; 58: 304–308 [33], and modified and reproduced (D) with permission from John D *et al*. JACC Cardiovasc Interv 2010; 3: 233–243 [34].

Early calcification can be clearly identified using von Kossa staining, appearing as finely stippled calcifications or small nodular concretions, typically located in the fibrosa, especially in areas with early atherosclerotic changes (Fig. 2A,B, Ref. [32]). The location is often specific and is seen along the “line of closure” just below the free margin of the aortic leaflets and at “hinge” points. These areas, forming a radial pattern near the attachment of the aortic root, are where early calcific deposits are found, representing regions prone to greater mechanical forces. Early calcifications gradually merge into larger, more complex nodules, extending toward the middle of the leaflet while sparing the free margin, and may eventually protrude onto the aortic surface. Calcification in each cusp has been observed in various forms, including bridges, fingers, and other patterns, such as “radiation” in radiographs (Fig. 3, Ref. [33, 34]) [33].

Aortic valve sclerosis (fibrosis) and calcification have been described to occur as age advances [35]. Stewart *et al*. [35] showed that in healthy individuals 65 to 74 years old undergoing echocardiography, aortic valve sclerosis was observed in 20% and calcification in 2%; in individuals 75 to 84 years old, sclerosis occurred in 35% and calcification in 2.4%; the highest was in individuals >85 years, with sclerosis observed in 48% and calcification in 4%. We have measured aortic valves from histologic sections, which show that valve thickness at the line of closure is 0.55 ± 0.11 mm in individuals 0–19 years of age, increasing to 1.12 ± 0.81 mm in individuals >65 years, and calcium was observed in 27.9% of valves. In late stages, calcification often develops on a thickened and fibrotic cusp and may even show focal bone formation with histologic evidence of bone matrices, osteocytes, osteoclasts, and marrow elements (Fig. 2C–H, Ref. [11]) [36]. In cases of severe calcific AS, gross examination reveals that valve cusps are significantly thickened and distorted by several calcium nodules, often filling the sinuses of the cusps involved, which ultimately compromises the function and integrity of the valve (Fig. 2D). Of note, calcification is often more prominent in the non-coronary cusps than the left and right coronary cusps, possibly as a result of relatively increased bidirectional and oscillatory shear stress in the non-coronary cusp.

## 5. Risk Factors

Previous epidemiological studies have identified several risk factors for calcific AS, many of which overlap with those for coronary atherosclerosis, including age, male sex, smoking, elevated cholesterol and lipoprotein(a) levels, hypertension, and metabolic syndrome [35, 37, 38, 39]. Furthermore, calcific AS has been linked to chronic kidney diseases and abnormalities in calcium and phosphate metabolism [40].

Since calcific AS and coronary artery disease (CAD) share common risk factors, CAD frequently coexists in patients with severe AS. The prevalence of significant CAD in severe AS patients ranges from 25–50% [41, 42]. CAD is reported in 35–65% of cases among those undergoing SAVR [43, 44] and in 40–75% of TAVR cases [41]. The impact of concomitant CAD on the prognosis of severe AS remains controversial. For SAVR, some studies have indicated higher long-term mortality in patients undergoing coronary artery bypass graft (CABG) surgery with SAVR compared to SAVR alone [45]. In contrast, a large observational study using propensity matching for comorbidities and risk factors found no significant difference in long-term mortality between these groups [44]. Regarding TAVR, while earlier studies suggested that the presence of CAD does not increase mortality rates [46, 47], recent findings indicate that the severity of CAD, rather than its presence alone, may influence outcomes of patients undergoing TAVR [48, 49]. Collectively, whether CAD directly affects the prognosis of patients with severe AS or serves as a bystander condition remains unresolved.

## 6. Pathophysiology of Calcific Aortic Stenosis

**Fig. 4.**
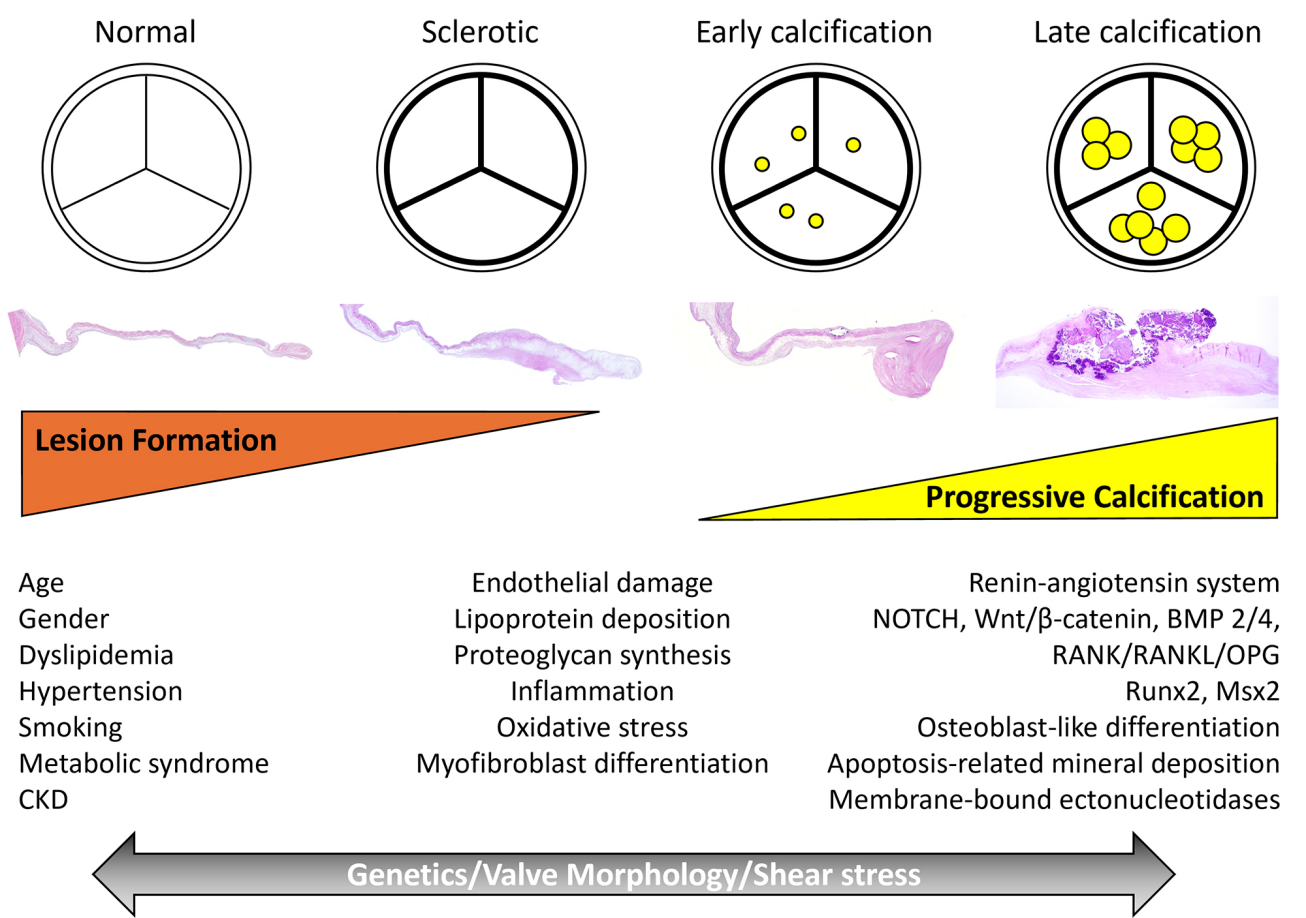
**Diagram Illustrating the Potential Paradigm for the Understanding of Calcific Aortic Stenosis**. Traditional risk factors promote valve sclerosis and calcification via similar mechanisms to atherosclerosis. Endothelial damage and proteoglycan synthesis allow for lipoprotein deposition, which contributes to inflammation and oxidized stress. These processes lead to myofibroblast differentiation and activate the renin-angiotensin system, leading to valve sclerosis. Pathways such as NOTCH, Wnt/β-catenin, BMP2/4, and RANK/RANKL/OPG, along with transcription factors like Runx2 and Msx2, drive the osteoblast-like differentiation of valvular interstitial cells. Apoptosis-related mineral deposition and membrane-bound ectonucleotidases further contribute to calcification, perpetuating the disease process. Genetic factors, valve morphology (e.g., unicuspid and bicuspid valves), and shear stress likely contribute to disease progression across the full spectrum of disease. BMP, bone morphogenetic protein; CKD, chronic kidney disease; Msx2, msh homeobox 2; OPG, osteoprotegerin; RANK, receptor activator of nuclear factor kappa-B (NF-κB); RANKL, receptor activator of NF-κB ligand; Runx2, runt-related transcription factor (RUNX) family of genes. Modified from Elena L *et al*. Future Cardiol 2011; 7: 629–642 [11].

Calcific AS was once regarded as a degenerative disease, both passive and age-related. However, recent studies have suggested that it is a disease involving active cellular processes, with identifiable clinical and genetic risk factors, as well as specific cellular and molecular pathways (Fig. 4, Ref. [11]). Indeed, similarities in clinical risk profiles and extensive experimental research revealed that aortic valve calcification involves mechanisms akin to atherosclerosis, including lipoprotein deposition, chronic inflammation, and mineralization.

Endothelial damage allows for lipid infiltration, specifically low-density lipoprotein and Lp(a), which triggers the recruitment of inflammatory cells [50]. This endothelial injury can be induced by several factors including lipid-derived species, cytokines, and mechanical stress [31]. Proteoglycans such as biglycan and decorin are highly expressed in early phases of calcific aortic valve disease, potentially playing a key role in lipid retention and modification [51, 52]. Additionally, the production of reactive oxygen species (ROS), which is enhanced by the uncoupling of nitric oxide synthase, promotes the oxidization of lipids with osteogenic properties [53, 54].

Inflammation, encompassing innate and adaptive responses, follows endothelial damage and oxidized phospholipid deposition, driving disease progression [55, 56]. Macrophages and T lymphocytes are key players in the inflammatory process; however, the involved immune cell network is highly complex, comprising a diverse and heterogeneous array of immune cell phenotypes [55]. Briefly, pro-inflammatory (M1) macrophages, which are the predominant subset found in calcific AS, produce pleiotropic cytokines, such as tumor necrosis factor (TNF)-α, interleukin (IL)-1β, and IL-6. TNF-α strongly activates the canonical nuclear factor kappa-B (NF-κB) pathway, which promotes the expression of genes associated with inflammation and affects the mineralization of VIC [57]. Additionally, both TNF-α and IL-6 contribute to extracellular matrix (ECM) remodeling, trigger endothelial-to-mesenchymal transition, increase expression of bone morphogenetic protein-2 (BMP-2), and promote osteogenic differentiation of VICs *in vitro* [57, 58, 59]. In contrast, anti-inflammatory (M2) macrophages release cytokine transforming growth factor (TGF)-β, which promotes myofibroblastic differentiation of VICs and contributes to calcification [16, 60]. T lymphocytic infiltrate is also observed in calcific aortic valve disease, accompanied by increased neovascularization and osseous metaplasia [61, 62]. Both CD8^+^ T cells and CD4^+^ T cells are infiltrated in aortic valves, with a predominance of CD4^+^ T cells [61, 63]. Furthermore, transcriptomic data from calcific AS showed that T lymphocytes, both in the valve and circulation, exhibit a clonal expansion, suggesting the proliferation of antigen-specific T cell repertoire that may or may not be in response to disease-related antigens [64, 65]. Angiotensin-converting enzyme (ACE) and chymase facilitate the production of angiotensin II, which enhances the synthesis and secretion of collagen by VICs [66, 67]. In addition, angiotensin II is a strong activator of the NF-κB pathway, leading to a significant fibrotic response [68].

The phase of mineralization largely involves two mechanisms: osteogenic differentiation of VICs and mineral deposition. Osteogenic differentiation occurs through several distinct pathways, including osteoprotegerin/RANKL (receptor activator of nuclear kappa B ligand) signaling, NOTCH1 signaling, BMP-2 signaling, Wnt/β-catenin pathways, and increased expression of runt-related transcription factor (RUNX) family of genes (*Runx2*) and Msh homeobox 2 (*Msx2*) [69, 70, 71, 72, 73, 74]. On the other hand, mineral deposition is characterized by the formation of a nidus for apoptosis-mediated calcification through apoptotic remnants from dysfunctional VICs and immune cells [31, 55, 72]. This mechanism can be further facilitated by membrane-bound ectonucleotidases [72, 75]. Valve calcification results in compliance mismatch, leading to increased mechanical stress and injury, which in turn, promotes further calcification through osteogenic differentiation and apoptosis. In this way, the development of calcific AS shares many similarities with the pathophysiology of atherosclerotic cardiovascular disease.

## 7. Etiology, Classification, and Histopathology of Congenital Valve Disease

### 7.1 Bicuspid Aortic Valve

BAV is one of the most common congenital heart diseases. Although it initially functions normally at birth, BAV undergoes a degenerative process similar to that of TAV. However, BAVs typically develop stenosis about 10–20 years earlier than TAVs [76]. BAV features a conjoint area of two underdeveloped leaflets that are joined together in the area of commissure. These leaflets are malformed and known as a “raphe”. Normally, the commissure of the aortic valve is the space where two adjacent leaflets attach parallelly without adhering to each other. In BAVs, one or two commissures may be obliterated, or a raphe may be absent [21].

The pathogenesis of congenital BAV formation remains unknown. Several researchers believe that abnormal blood flow during valvulogenesis leads to improper separation of the valve cusp [77], although this claim lacks sufficient evidence. BAV is widely accepted to have a genetic basis and is hereditary within families, occasionally associated with genetic syndromes such as Marfan syndrome, Turner syndrome, and Loeys-Dietz syndrome [78, 79]. However, BAV is a complex disorder with a polygenic basis, exhibiting incomplete penetrance and variable expressivity [80]. To date, variants in *NOTCH1*, *MYH6*, *GATA4*, *GATA5*, *GATA6*, *PALMD*, *EXOC4*, *TEX41*, *FBN1*, *ROBO4*, and *SMAD6* have been reported to be associated with BAV [81, 82, 83, 84, 85, 86, 87, 88]. BAV is also strongly associated with congenital abnormalities of the aorta, such as coarctation and patent ductus arteriosus, as well as the proximal coronary vasculature. Additionally, after development, it could be linked to conditions like aortic dilation, aneurysm, and dissection. In this context, BAV should be recognized as a condition that impacts the entire aortic root.

**Fig. 5.**
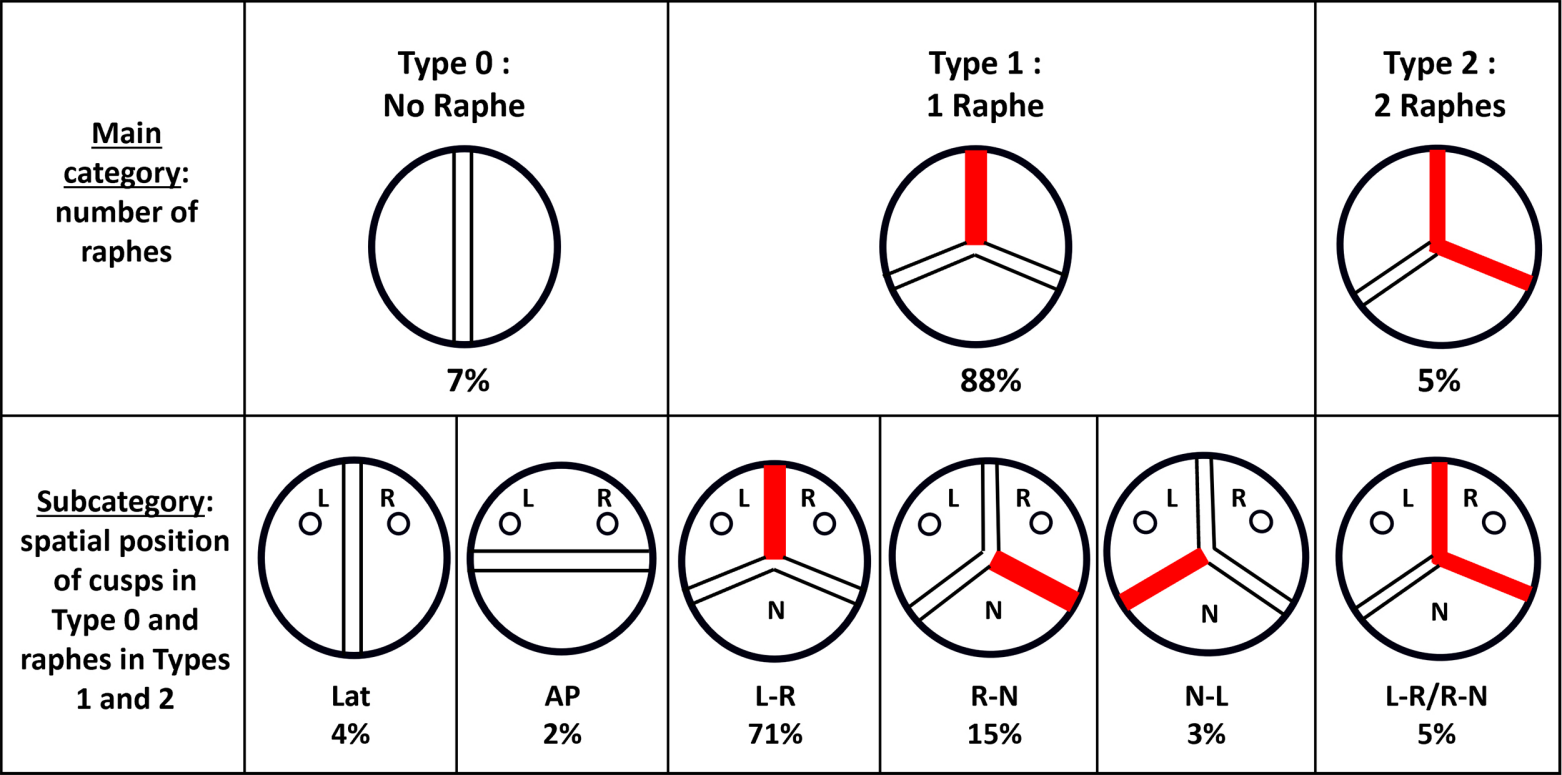
**Schematic Overviews of Bicuspid Aortic Valve (BAV)**. Red lines in the schematic illustrations represent a raphe. BAV is classified into three main types based on the number of raphes: Type 0, with no raphe (7%); Type 1, the most common type of configuration, with one raphe (88%); and Type 2, with two raphes (5%). Each type is further categorized as AP, Lat, L-R, R-N, N-L, and L-R/R-N. AP, anterior-posterior; Lat, lateral; L, left coronary sinus; R, right coronary sinus; N, non-coronary sinus. Modified and reproduced with permission from Sato Y *et al*. Mastering Structural Heart Disease (pp.12). 1st edn. Wiley: NJ. 2023 [32].

BAVs are categorized into three main types based on their morphologic characteristics: types 0, 1, and 2 (Fig. 5, Ref. [32]) [89]. Sievers *et al*. [89] demonstrated the prevalence of BAV types from 304 patients undergoing surgery. The classification is based on: (1) the number of raphes, (2) the spatial positioning of cusps or raphes, and (3) the functional status of the valve (i.e., stenosis, regurgitation, or both). Type 0, found in 7% of cases, is considered purely bicuspid without a raphe, with commissures located either anterior/posterior or left/right. Type 1, which is the most common (88%), consists of three developmental cusps and two commissures instead of three. In this type, two of the cusps are unequal in size, with the larger conjoint cusp featuring a central raphe. The conjoint cusp is generally less than twice the size of the non-conjoint cusp. The most prevalent common type is the fusion of the left and right coronary cusps (71%), followed by fusion of the non-coronary and right coronary cusps (15%), and the least common is fusion of the left and non-coronary cusps (3%). According to Sievers’ classification, BAVs with two raphes are categorized as type 2 (5%), while several other studies categorize them as unicommissural or unicuspid valves [90, 91].

**Fig. 6.**
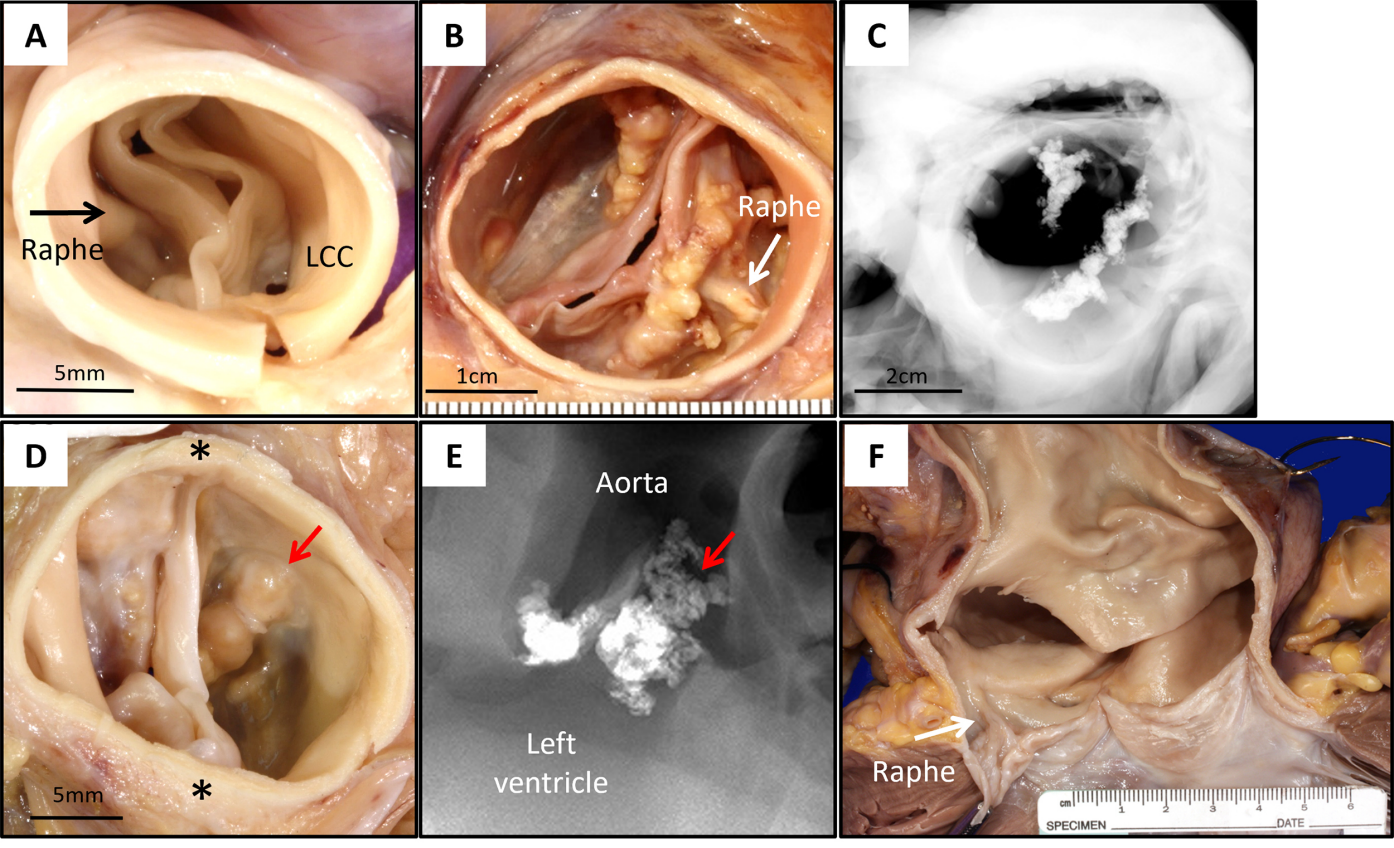
**Representative Images of BAV**. (A) Aortic valve from a 5-year-old male who experienced sudden death from unknown causes. Gross image from the aortic surface (A) shows BAV with a midline raphe between conjoint right and non-coronary cusps (black arrow). The conjoint cups are thickened and stiff with prominent raphe, without any calcification, scale bar: 5 mm. (B,C) Aortic valve from a 47-year-old male who experienced sudden death during motocross. (B) Gross image from aortic surface shows conjoint left and right coronary cusps, anterior calcified raphe (white arrow), and calcification of the aortic side of the non-coronary cusp near the left commissure, scale bar: 1 cm. (C) Radiography shows calcification corresponding to the gross image, scale bar: 2 cm. (D,E) Aortic valve from a 53-year-old male who experienced sudden death from unknown cause. (D) Gross image from the aortic surface shows calcific aortic stenosis arising in type 0 BAV: left and right coronary valve cusps with anterior-posterior commissures (*), no well-defined raphe, scale bar: 5 mm. (E) Radiography shows severe calcification corresponding to gross images (red arrows). (F) Gross image of the aortic valve and ascending aorta from a 38-year-old male who experienced sudden death from ruptured ascending aortic aneurysm. Note BAV with raphe (white arrow) between conjoint right and left coronary cusps is seen in (F). LCC, left coronary cusp.

BAVs often exhibit signs of calcification by the time individuals reach their thirties [92]. Calcification typically initiates in the raphe, appearing as a linear opacity on radiographs, and gradually extends toward the free margin of the leaflet, generally sparing the true commissures (Fig. 6). In severe AS cases related to BAVs, calcification spreads diffusely through the conjoint and non-conjoint cusps, involving the body of the leaflets. Calcific nodules potentially ulcerate the aortic surface (Fig. 6). The variability of the raphe in BAVs sometimes complicates the distinction between congenital and acquired BAV (e.g., rheumatic AS). In congenital BAVs, the raphe typically contains abundant elastic fibers, whereas acquired BAVs show collagen-rich fibrous tissue at fused commissures due to inflammatory scaring [93].

### 7.2 Unicuspid Aortic Valve

**Fig. 7.**
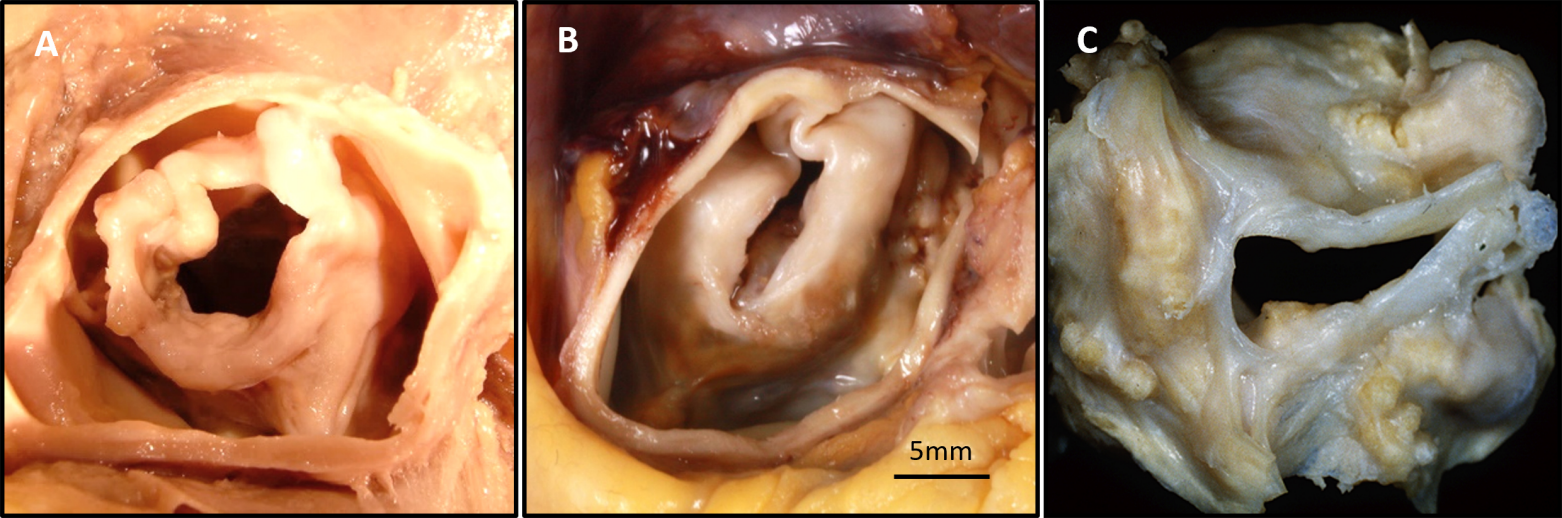
**Representative Images of Unicuspid Aortic Valve (UAV)**. (A) UAV from a 47-year-old male who experienced sudden death from unknown causes. Gross images from the aortic surface shows dome-shaped UAV with three raphes, diffuse nodular calcification, an aortic orifice 1 cm in diameter, and concentric left ventricular hypertrophy. (B) UAV from a 28-year-old male who experienced sudden death from unknown causes. Gross image from the aortic surface shows calcific UAV with single left lateral commissure, right lateral, and anterior raphes, scale bar: 5 mm. (C) Surgically removed unicommissural aortic valve (AV).

UAVs are classified into two morphologic types: (1) an acommissural valve shaped like a dome with three aborted commissures (or raphes) and (2) a unicommissural valve, characterized by a slit-like opening extending through the aortic wall, with a single intact commissure (Fig. 7) [27]. The acommissural form is typically accompanied by left heart failure symptoms that develop early in life, whereas the unicommissural form has a less-aggressive course, as the presence of a commissure results in a relatively larger valvular orifice area compared to the acommissural form. Unicommissural unicuspid aortic valve accounts for 60% of AS cases in patients under 15 years of age [23]. Leaflet dysplasia is frequently observed, with varying degrees of leaflet calcification; however, dysplasia in UAVs is generally more severe compared to BAVs, and its severity is influenced by the patient’s age [30].

### 7.3 Rheumatic Valve Disease

Rheumatic heart disease occurs due to valvular damage triggered by an abnormal autoimmune response to a group A streptococcal infection, typically during childhood [94]. The use of penicillin as a preventive measure is highly effective and, in developed countries, has nearly eliminated rheumatic heart disease [95]. Nevertheless, this disease remains the leading cause of heart failure in children and young adults, resulting in at least 200,000–250,000 premature deaths every year in emerging nations [96]. A global cohort including 14 developing countries reported that rheumatic heart disease was twice as common among females, with a median age of 28 years [97].

The precise pathophysiology of the disease is not fully understood, but previous reviews have indicated that the main mechanism involves antigenic mimicry combined with an abnormal immune response from the host [98]. This process is based on three key factors: the presence of rheumatogenic group A streptococcal strains, a genetically susceptible host, and an abnormal immune response from the host. Genetically, human leukocyte antigen (HLA) class II molecules have been linked to an increased risk of the disease, although no single HLA haplotype or combination has consistently been tied to disease susceptibility [99]. CD4^+^ T cells are key players responding to cross-reactive antigens from streptococcal strains, which produce Th1 and potentially Th17 cytokines, leading to further inflammatory response in the heart valves [100, 101].

**Fig. 8.**
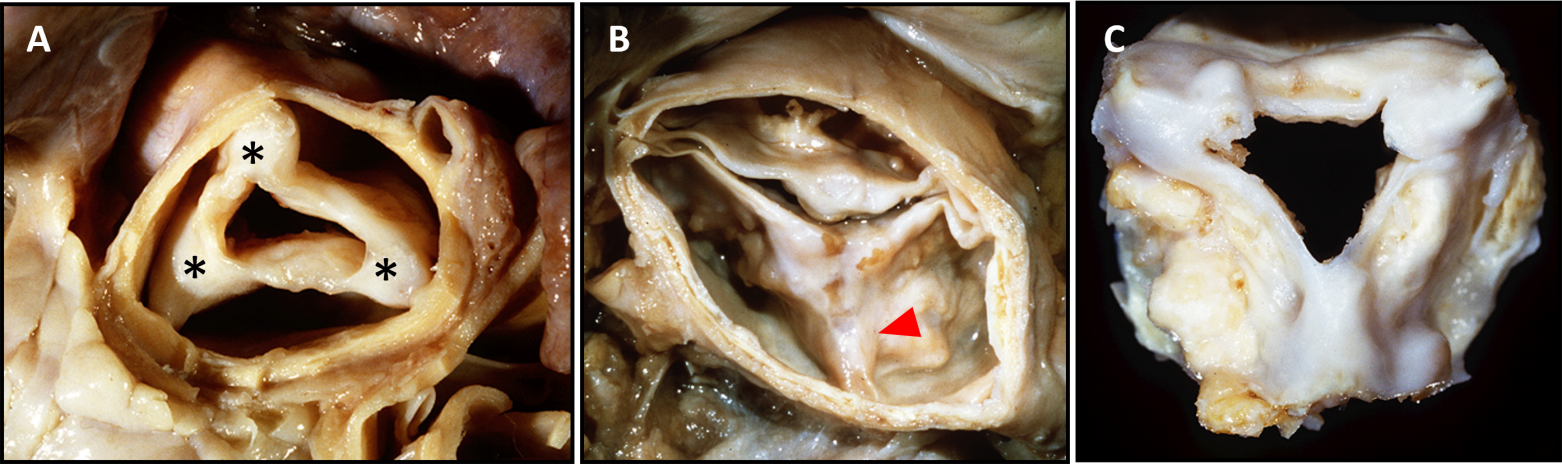
**Representative Images of Post-Rheumatic Aortic Valve Diseases**. (A) A 44-year-old male with known heart disease was found dead. At autopsy, he had an enlarged heart with severe mitral stenosis and aortic stenosis. Note fusion of all three aortic valve commissures (*), thickening and fibrosis of all three aortic leaflets, and no calcification. (B) An acquired bicuspid stenotic aortic valve was found in this 65-year-old man who died while awaiting valve replacement. The anterior commissure is fused (red arrowhead), and this commissure is at the same level as and equidistant from the other two non-fused commissures. (C) A surgically excised aortic valve in a 50-year-old female with mitral and aortic stenosis. All three commissures are fused, and cusp fibrosis and calcification are evident with calcification present both in the commissures and leaflets. Reproduced with permission from Virmani R *et al*. Cardiovascular pathology (pp. 254). 2nd edn. W.B. Saunders Company: Philadelphia, 2001 [9].

Typically, rheumatic heart disease affects multiple valves. The most common pattern is a combination of aortic and mitral valve disease, followed by mixed mitral valve disease. Isolated aortic valve disease is rare (2–10%) [97, 102, 103]. In contrast to bicuspid AS and senile AS, rheumatic AS has relatively little calcification [21]. Cuspid thickening and commissural fusion of at least one and generally two or three commissures are the hallmark of this disease (Fig. 8, Ref. [9]) [104, 105].

When conducting valve replacement surgery, the selection of prosthesis (whether bioprosthetic or mechanical) requires careful consideration, taking into account the patient’s age, potential for pregnancy, and the likelihood of adherence to anticoagulant therapy, particularly in remote or socioeconomically disadvantaged areas [106].

## 8. Management of AS

The only curative treatment available for patients with symptomatic severe AS is the implantation of a prosthetic heart valve, either surgically or percutaneously. Traditionally, the choice between TAVR and SAVR was often straightforward, based on age, anatomy, and surgical risk. However, current recommendations emphasize a more comprehensive approach, considering multiple factors when choosing a prosthetic valve, including the severity, symptoms, left ventricular function, comorbidities, frailty, cognitive function, and patients’ preferences [107, 108].

Specifically, in current American College of Cardiology (ACC)/American Heart Association (AHA) guidelines, SAVR is recommended for patients who are <65 years of age or have a life expectancy >20 years who require AVR, whereas transfemoral TAVR is recommended for patients >80 years of age or those with a life expectancy <10 years, and either TAVR or transfemoral TAVR is recommended for patients who are 65 to 80 years of age without anatomic contraindication to TAVR, after shared decision-making (class IA) [107]. On the other hand, in current European Society of Cardiology (ESC)/European Association for Cardio-Thoracic Surgery (EACTS) guidelines, SAVR is recommended in younger patients who are low risk for surgery (<75 years and The Society of Thoracic Surgeons (STS)-Predicted Risk of Mortality (PROM)/EuroScore II <4%), TAVR is recommended in older patients (≥75 years) or in those who are high risk (STS-PROM/ EuroScore II >8%), and either SAVR or TAVR are recommended for remaining patients according to individual clinical, anatomical, and procedural characteristics (class IA) [108]. SAVR is generally recommended for younger patients primarily due to concern about the durability of the TAVR valves. Additionally, when opting for SAVR, mechanical heart valves are often preferred for younger patients because bioprosthetic heart valves tend to have limited durability, though they have the advantage of not requiring lifelong anticoagulants.

Moreover, both the ACC/AHA and the ESC/EACTS guidelines suggest that SAVR is more appropriate for BAV cases [107, 108]. However, a recent randomized trial showed that among low-risk patients aged ≤75 years with severe symptomatic AS, the rate of all-cause death, stroke, and rehospitalization at 1 year was comparable between SAVR (7.1%) and TAVR (10.2%), although the study was underpowered due to its small sample size [109].

Although no medical therapies are currently approved, several novel treatment strategies have been explored based on an advanced understanding of the underlying pathophysiological mechanisms [110]. These include therapies targeting lipoprotein(a), such as niacin, antisense oligonucleotide therapy, and proprotein convertase subtilisin/kexin type 9 (PCSK9) inhibitors; bisphosphonates targeting the receptor activator of nuclear factor kappa-B (NF-κB)/receptor activator of NF-κB ligand/osteoprotegerin (RANK/RANKL/OPG) pathway; vitamin K as a cofactor in the activation of matrix-Gla proteins; and therapies targeting the nitric oxide pathway—such as nitrates, ataciguat, and phosphodiesterase inhibitors. Dipeptidyl peptidase-4 (DPP-4) inhibitors, which target osteogenic differentiation of VICs via modulation of insulin-like growth factor-1 signaling, and anti-inflammatory agents like colchicine have also shown potential. Collectively, while several promising therapeutic targets have been identified and are under investigation, further research is required to translate these findings into clinical practice.

## 9. Long-Term Durability of SAVR and TAVR Valves

Previous analyses of randomized control trials or propensity score-matched analyses from registries comparing TVAR vs. SAVR revealed assuring results on the midterm durability of TAVR, which are favorable when compared to SAVR [111]. The incidence of all-cause TAVR bioprosthetic valve failure was as follows: (i) PARTNER 2A and SAPIEN 3 intermediate-risk registry: 4.7% in TAVR with SAPIEN XT, 2.6% in TAVR with SAPIEN 3 vs. 1.3% in SAVR at 5 years [112]; (ii) PARTNER 3: 3.3% in TAVR with SAPIEN 3 vs. 3.8% in SAVR at 5 years [113]; and (iii) NOTION RCT: 9.7% in TVAR with CoreValve vs. 13.8% in SAVR at 10 years [114]. Therefore, with the exception of the first generation of SAPIEN valves, the midterm (up to 7–8 years) durability of TAVR valves is at least comparable to that of SAVR valves. The NOTION RCT is the only trial that has reached 10-year follow-up. Although the results of this trial are quite promising, no definitive conclusion can be made on the long-term durability of TAVR based on these findings, because this trial had several limitations: one was that only 25% of the patients remained alive after 10 years; the other was that the TAVR arm only included first-generation valves, and the SAVR arm included 35% of the Trifecta (Abbott) or Mitroflow (Sorin) valves, which have both been shown to have durability concerns. The long-term durability of TVAR valves will need to be confirmed by analyses of the low-risk TAVR vs. SAVR trials at 10 years [113, 115].

**Fig. 9.**
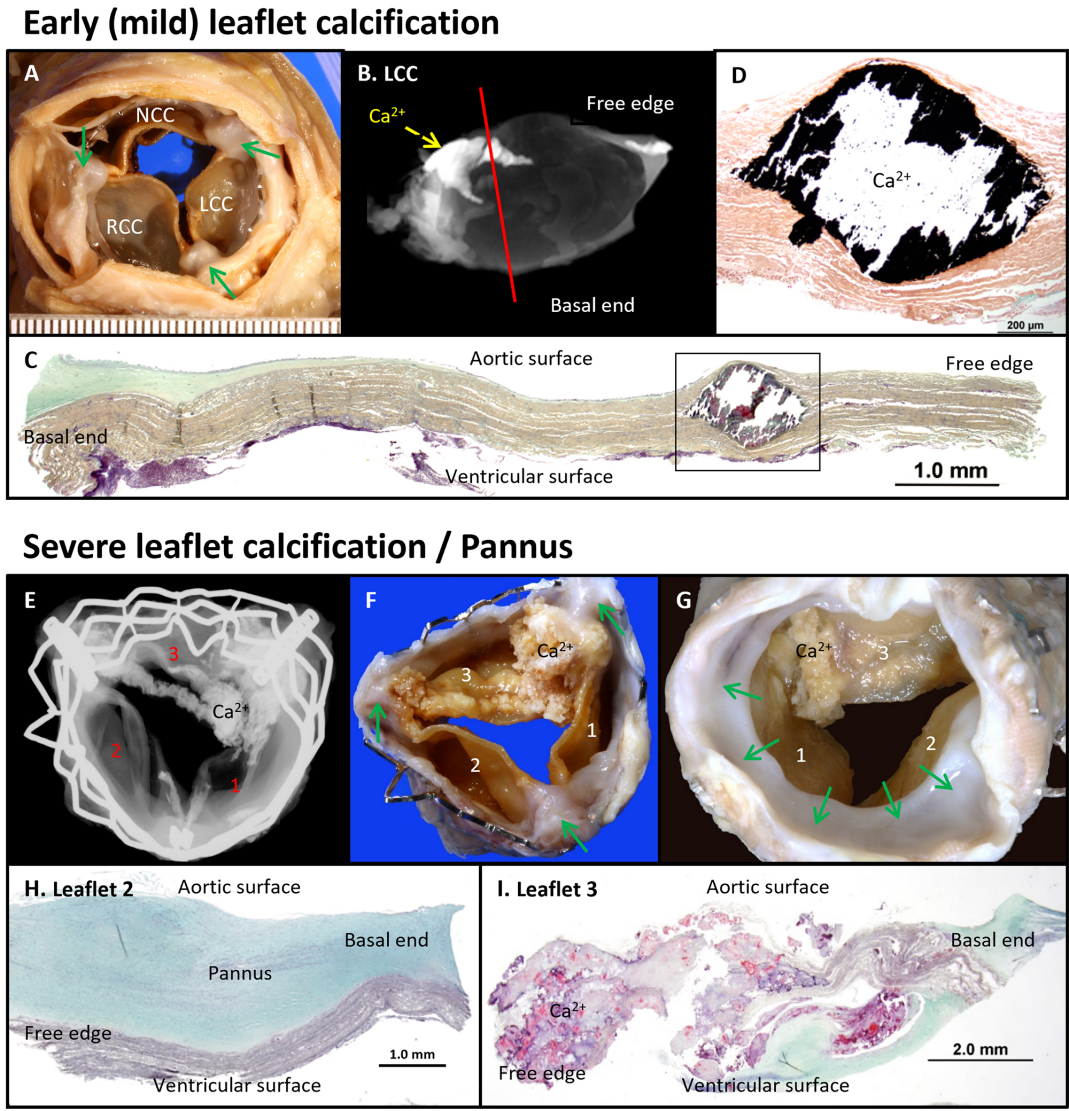
**Calcification and Pannus Formation in Edwards SAPIEN TAVR Leaflets**. Early leaflet calcifications observed four years after implantation (A–D). (A) Gross image from the aortic surface showing commissural fusion (green arrows). (B) Radiographic image highlighting focal calcification (Ca^2+^) at the commissure site. (C) Histologic section showing focal intrinsic calcification in the valve leaflet (Movat Pentachrome stain), scale bar: 1.0 mm. (D) High-power image of the black boxed area in D (von Kossa stain), scale bar: 200 µm. Severe leaflet calcification with pannus formation observed in a surgically removed TAVR valve five years following the implantation (E–I). (E) Radiograph showing severe leaflet calcification (Ca^2+^), predominantly at the commissure sites between leaflet 1 and 3. (F,G) Gross images from the aortic and ventricular surfaces, respectively, with green arrows showing pannus formation, mainly on the ventricular surface. (H) Histologic section revealing a thick pannus composed of smooth muscle cells in a proteoglycan (green) collagenous matrix on leaflet 2, scale bar: 1.0 mm. (I) Severe calcification with neointimal growth in leaflet 3, scale bar: 2.0 mm. Modified and reproduced with permission from Yahagi K *et al*. Catheter Cardiovasc Interv 2017; 90: 1048–1057 [118]. NCC, non-coronary cusp; RCC, right coronary cusp.

**Fig. 10.**
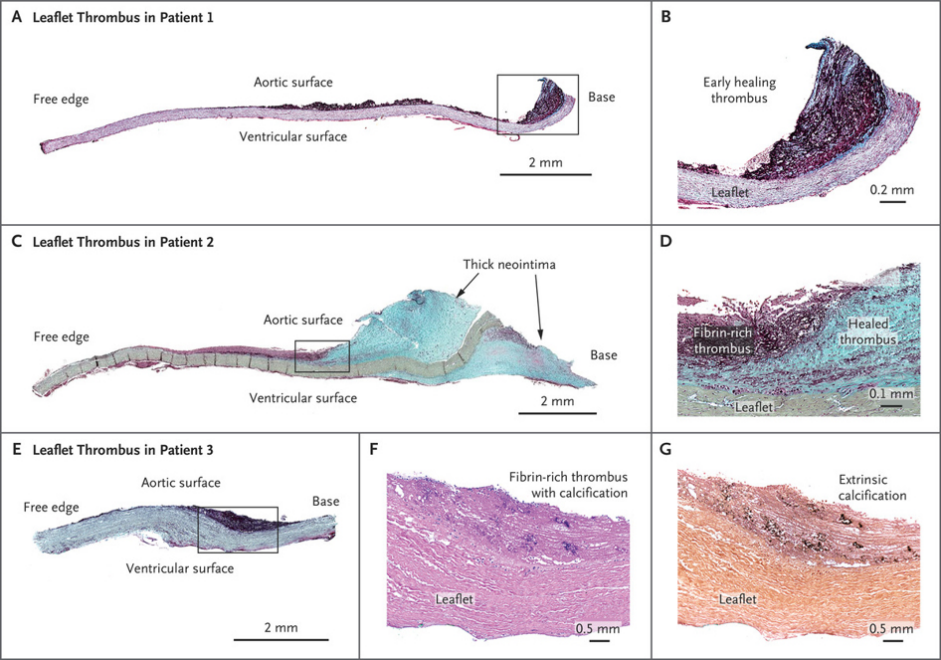
**Histological Images of Leaflet Thrombus from Patients Who Underwent TAVR**. (A) Low-power image of a leaflet with fibrin-rich thrombus on the aortic surface, 38 days after implantation, scale bar: 2 mm. (B) High-power image of the boxed area in (A) showing the thrombus attached to the base of the leaflet, with early thrombus organization indicated by visible proteoglycan (green areas within the magenta-colored thrombus), scale bar: 0.2 mm. (C) Low-power image of a leaflet at 105 days after implantation, showing fibrin-rich thrombus in the midportion and a thickened neointima from healed thrombus at the base, on both the aortic and ventricular surfaces, scale bar: 2 mm. (D) High-power image of the boxed area in (C), showing organizing thrombus, with fibrin-rich thrombus transitioning to smooth muscle cells in a proteoglycan matrix (green), scale bar: 0.1 mm. (E) Low-power image of a leaflet at 517 days after implantation showing fibrin-rich thrombus on the aortic surface, scale bar: 2 mm. (F) High-power image of the leaflet thrombus, with purple areas indicating calcification (H&E stain), scale bar: 0.5 mm. (G) Early spotty extrinsic calcification in the thrombus (von Kossa stain), scale bar: 0.5 mm. All specimens in panels (A–E) are stained with Movat Pentachrome. TAVR, transcatheter aortic valve replacement. Reproduced with permission from Yahagi K *et al*. N Engl J Med 2020; 383(2): e8 [124].

We have recently reported our findings on bioprosthetic valve dysfunction in SAVR and TAVR valves that were removed surgically or at autopsy. Bioprosthetic valve dysfunction means impaired functional performance of the bioprosthetic valve, encompassing four types: structural valve deterioration (SVD), non-SVD, thrombosis, and endocarditis [108, 116, 117]. Among these, SVD is the most prevalent cause of failure, and is characterized by irreversible changes in the bioprostheses due to leaflet thickening, fibrosis, pannus formation, calcification, and leaflet tear [111]. We previously published on 43 TAVR cases [118, 119] from the PARTNER trial [120] as well as the CoreValve U.S. Pivotal High-Risk Trial [121]. In two cases of Edwards SAPIEN valves with bovine pericardial leaflets, valve calcification was observed (Fig. 9, Ref. [118]). In addition, valve leaflet thrombosis is one of the most important causes of bioprosthetic valve dysfunction, which can be observed as “hypo-attenuated leaflet thickening (HALT)” by computed tomography. Subclinical leaflet thrombosis may occur in as much as 5 to 25% of patients during the first year following TAVR or SAVR [122, 123]. Valve thrombi observed histologically within 30 days are primarily platelet-rich, whereas those seen after 30 days are predominantly fibrin-rich, with or without signs of organization. These later-stage thrombi show the presence of smooth muscle cells within a proteoglycan- and type III collagen-rich matrix (neointima formation) (Fig. 10, Ref. [124]) [118, 119].

## 10. Conclusions

AS is an age-related disease and previous clinical trials have identified age as the most significant risk factor associated with aortic stenosis or sclerosis [35]. However, recent studies have revealed that calcific AS is not merely a “degenerative” disease caused by time-dependent wear and tear of the leaflets Instead, it is now considered to involve active cellular mechanisms including lipoprotein deposition, chronic inflammation, and mineralization, similar to that of atherosclerotic disease progression. These processes could potentially be targeted by medical treatments; however, to date, no pharmacological treatments are able to successfully halt the progression of AS or improve long-term outcomes. As a result, treatment is currently limited to SAVR or TAVR, which can be too invasive for elderly patients.

The onset and progression of AS varies significantly depending on the types of AS. Congenital AS (e.g., bicuspid and unicuspid AS) and rheumatic AS tend to develop at a younger age. In contrast, degenerative AS in tricuspid valve typically occurs at an older age. Given the aging population and increased life expectancy, the need for treatment of symptomatic AS in elderly patients is growing and is expected to continue increasing without effective medical treatment. TAVR has become an important, minimally invasive option for elderly AS patients; however, its long-term outcome remains to be fully elucidated. A key concern is the durability of TAVR valves over time, as bioprosthetic valve dysfunction can occur due to various mechanisms. Understanding the underlying causes and mechanisms is crucial for making appropriate treatment choices (i.e., valve selection and pharmacological therapy) and essential for effective lifetime management of AS.
